# Optimizing Marine eDNA Protocols for Rare Megafauna: A Case Study on Dugongs in the Western Indian Ocean

**DOI:** 10.1002/ece3.74500

**Published:** 2026-10-16

**Authors:** Lea Bernagou, Auriane Serval, François Beudard, Jerome Mathey, Yann Mercky, Charles Le Bozec, Evan Trotzuk, Keila Paunde, Marielle Garcia, Eva Bellemain, Emmanuel Corse

**Affiliations:** ^1^ Association des Naturalistes Environnement et Patrimoine de Mayotte Dembéni Mayotte; ^2^ DroneGo (SAS) Tsingoni Tsingoni Mayotte; ^3^ Université de Mayotte Dembeni France; ^4^ African Parks, Bazaruto Archipelago National Park Bazaruto Mozambique; ^5^ ARGALY (SAS) Sainte‐Hélène‐du‐Lac France; ^6^ MARBEC, CNRS, Ifremer, IRD Université de Montpellier Montpellier France

**Keywords:** biomonitoring, diagnostic PCR, eDNA detectability, marine megafauna, proof‐of‐concept, qPCR assay development, rare species, sirenians

## Abstract

Assessing habitat use, spatial distribution, and site fidelity is critical for the conservation of threatened marine species, yet monitoring rare and elusive species in these types of complex environments remains difficult. This limitation is particularly critical for dugong ( *Dugong dugon* ) populations in the Western Indian Ocean, which are extremely small, fragmented, and difficult to monitor, posing a major conservation challenge. This pilot study aimed to develop a cost‐effective and field‐optimized eDNA workflow based on species‐specific qPCR for large‐scale monitoring of this threatened sirenian in shallow tropical coastal ecosystems. Field sampling was conducted in Mozambique, which hosts the only remaining large dugong population in the region. Using a species‐specific qPCR assay independently developed and validated for this study, we evaluated how sampling design and sample processing influence dugong eDNA detection under natural conditions. Sampling was conducted along survey tracks using different sampling and DNA extraction strategies to evaluate three key variables of the detection protocol—(i) vertical sampling position (surface, near‐bottom, and sediment), (ii) filtration volume (1.5, 30, and 60 L), and (iii) field versus laboratory DNA extraction—resulting in a total of 11 sampling–extraction strategies. At every visually confirmed location, at least one of the 11 sampling–extraction strategies resulted in a positive detection, including up to 2 h after the last visual sighting. All strategies detected dugong DNA except those based on sediment sampling. qPCR detection was influenced by an interaction between filtration volume and vertical sampling position: at low filtration volume (1.5 L), detection was higher near the bottom than at the surface, whereas this difference disappeared at 60 L. Conversely, increasing filtration volume improved detection at the surface but had little effect near the bottom. Laboratory extractions markedly outperformed field extractions. The sampling strategy yielding the highest detection rate—large‐volume near‐bottom filtration followed by laboratory‐based sample processing—detected dugong DNA in all cases where a visual sighting had occurred. Overall, these results show that filtration volume can partly compensate for differences in detection associated with vertical sampling position and provide a robust and field‐applicable framework for eDNA‐based dugong monitoring, supporting scalable and long‐term surveillance efforts in tropical coastal ecosystems.

## Graphical abstract

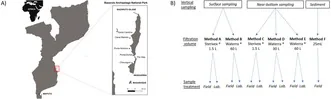

**Abbreviations**
BANP: Bazaruto Archipelago National Park
eDNA: Environmental DNA
qPCR: quantitative Polymerase Chain Reaction

## 1 Introduction

Since the emergence of environmental DNA (eDNA) approaches, aquatic biodiversity monitoring has been transformed by enabling non‐invasive detection of species presence over ecological timescales of hours to days (e.g., Ficetola et al. 2008). As a result, eDNA has proven particularly effective for detecting rare and elusive species that are difficult to monitor using conventional survey methods (Jerde et al. 2011; Bonfil et al. 2021; Meulenbroek et al. 2022). Over the past decade, rapid technological advances have accelerated the transition toward high‐throughput and large‐scale monitoring workflows, improving scalability while reducing operational constraints. These developments have been driven by three major and interconnected objectives: optimizing analytical performance, reducing costs, and ensuring operational feasibility under real field conditions and promoting standardized workflows to enhance reproducibility and long‐term data usability (e.g., Smart et al. 2016; Thomas et al. 2019; Shea et al. 2023; Lewis et al. 2026).

First, optimizing analytical performance is essential for maximizing detection reliability. The success of any eDNA survey depends strongly on the optimization of both field and laboratory procedures (e.g., Hinlo et al. 2017), particularly in marine environments where DNA persistence and detection are constrained by microbial degradation, physicochemical stressors, and dilution in dynamic systems (Ballmer et al. 2024; Xiong et al. 2025), as well as the rapid degradation of DNA under tropical UV‐exposed marine conditions (Lamb et al. 2022). Accordingly, conducting pilot studies is considered a critical step to assess protocol performance and validate proof of concept before broader deployment (Goldberg et al. 2016). Key field parameters—including filtration strategy, filtered water volume, vertical sampling position, and preservation method—have all been shown to critically influence detection probability (Pilliod et al. 2013; Barnes et al. 2014; Moyer et al. 2014; Hunter et al. 2019; Cowart et al. 2022; Jensen et al. 2024) and therefore represent adjustable variables that can be tested during pilot phases. Second, cost‐effective analytical approaches are required to enable large‐scale implementation. Diagnostic PCR‐based methods allow sensitive detection of specific target taxa without sequencing and, when implemented with commonly used methods such as quantitative PCR (qPCR), provide high sensitivity at relatively low cost, making them particularly suitable for broad monitoring and scalable applications (Ficetola et al. 2008; Wilcox et al. 2013; Wang et al. 2019; Andruszkiewicz et al. 2020). Finally, effective eDNA workflows must be compatible with field constraints. Field‐friendly eDNA approaches aim to balance detection efficiency with practical considerations by minimizing handling complexity, filtration time, hazardous reagent use, and consumable dependency, while ensuring DNA stability during transport and storage (Allison et al. 2021; Tomke et al. 2025). While most efforts have focused on improving water collection and filtration, the feasibility of performing DNA extraction directly in the field remains largely unexplored. Field‐based extraction could substantially reduce sample handling, cold‐chain dependence, and transport constraints, particularly in remote marine environments. However, its performance and the trade‐offs between logistical simplicity and DNA recovery efficiency remain poorly evaluated.

Dugongs ( *Dugong dugon* ) are large, primarily herbivorous marine mammals of high conservation concern whose populations have dramatically declined throughout their ranges (Marsh et al. 2025). Monitoring their habitat use is essential to guide conservation actions, yet conventional approaches based on visual or aerial surveys are constrained by the species' low densities, solitary, and cryptic behavior, and avoidance of human activity. In the Western Indian Ocean (WIO), dugongs persist in small, isolated populations, with the largest known subpopulation found around the Bazaruto Archipelago in Mozambique (Trotzuk et al. 2022). Monitoring these populations is particularly urgent, as they often consist of only a few individuals, making them highly vulnerable to extinction while also representing critically important units for the conservation of the species in the region (Plön et al. 2019).

eDNA‐based methods offer a non‐invasive alternative to conventional dugong surveys. Dugong DNA has previously been detected through metabarcoding (Sigsgaard et al. 2020), and the recent development of a species‐specific qPCR assay has further demonstrated the feasibility of targeted eDNA detection for *D. dugon* (Mousavi‐Derazmahalleh et al. 2026). However, translating analytical detection capability into reliable field detection remains challenging, particularly for small populations generating weak and spatially heterogeneous eDNA signals. For large and mobile species such as dugongs, this challenge is compounded by a mismatch between the broad spatial scale of daily movements and the small spatial footprint of typical water samples. Individuals may use seagrass meadows extending over several square kilometers while only transiently occupying specific locations, resulting in theoretically patchy eDNA signals. In addition, tidal renewal and water circulation may rapidly dilute or disperse eDNA, potentially erasing traces of recent presence and raising fundamental questions about appropriate sampling strategies. Thus, even with a sensitive species‐specific molecular assay, it remains unclear whether the passage of an individual through an extensive seagrass habitat leaves a sufficiently detectable eDNA signal, and how sampling design may influence its recovery.

Sampling design is therefore likely to play a key role in detection probability. Increasing filtration volume has been shown to enhance detection success, consistent with broader evidence that in situ filtration of large water volumes improves the detection of rare taxa in heterogeneous marine environments (Barnes et al. 2014; Hunter et al. 2018, 2019). Similarly, sampling strategies that integrate multiple points or survey tracks across the habitat may better capture spatially heterogeneous eDNA signals than isolated point samples. Beyond horizontal spatial heterogeneity, the vertical distribution of eDNA in the water column also represents an important but often overlooked dimension. In coastal marine environments, most eDNA studies rely on surface or near‐surface sampling, implicitly assuming that vertical mixing homogenizes eDNA concentrations between surface and near‐bottom waters. Under this assumption, surface and near‐bottom sampling would provide comparable detection probabilities. Alternatively, if vertical mixing is limited, eDNA may remain vertically structured, with different concentrations between surface and near‐bottom waters. In addition, benthic eDNA may accumulate near the bottom or become incorporated into surface sediments, where locally anoxic conditions promote longer‐term DNA persistence (Morard et al. 2011; Torti et al. 2018; Hoshino and Inagaki 2024; Jensen et al. 2024), making sediment a potentially valuable matrix for reconstructing recent dugong presence.

This study aimed to develop an optimized eDNA protocol for monitoring dugong presence in tropical marine environments, with particular emphasis on field sampling and sample‐processing strategies suitable for large‐scale deployment. In this context, our work was conceived as a pilot study to assess how methodological choices made upstream of molecular detection influence the recovery of rare dugong eDNA signals under natural conditions. A species‐specific qPCR assay was independently developed and validated for this study and subsequently used as the analytical tool for a field experiment conducted in Bazaruto Archipelago National Park (BANP). Rather than focusing primarily on assay development, the field experiment was designed to evaluate key components of the eDNA workflow that may determine detection success and operational feasibility under field conditions. Specifically, we assessed:

1. Vertical sampling position and benthic matrix: evaluation of surface and near‐bottom water sampling, together with sediment as an additional benthic matrix. We hypothesized that eDNA would be more concentrated in near‐bottom waters associated with dugong feeding activity than in surface waters, resulting in higher detection rates in near‐bottom samples.
2. Filtration volume: assessment of the effect of filtered volume using cartridges offering different filtration capacities, to improve detection of low‐concentration DNA; we hypothesized that larger filtered volumes increase the probability of capturing rare eDNA molecules, resulting in higher and more consistent detection rates compared to smaller‐volume samples.
3. Sample treatment: comparison of field‐ and laboratory‐based DNA processing in terms of DNA recovery, detection performance, and operational feasibility, considering logistical constraints and the need to avoid transporting hazardous reagents. We hypothesized that the field‐compatible extraction protocol would be sufficiently robust to provide DNA recovery and detection performance comparable to laboratory‐based processing.

## 2 Material and Methods

### 2.1 Field Sampling

During the October 2024 field campaign conducted in BANP, 12 non‐linear boat survey tracks were performed at an average speed of ~1.5 knots within known high‐use areas of dugong occurrence (Figure 1). Along each non‐linear survey track, different eDNA sampling methods were conducted using two different cartridges according to the target filtration volume: a polyethersulfone Waterra 0.45 μm filter cartridge (Calgary, Canada) used for large volume (60 and 30 L), and the polyethersulfone Millipore 0.22 μm Sterivex filters used for low volume (1.5 L): (A) 1.5 L of surface water filtered; (B) 60 L of surface water filtered; (C) 1.5 L of near‐bottom water filtered; (D) 30 L of near‐bottom water filtered; (E) 60 L of near‐bottom water filtered; (F) approximately 25 mL of sediment collected.

**FIGURE 1:**
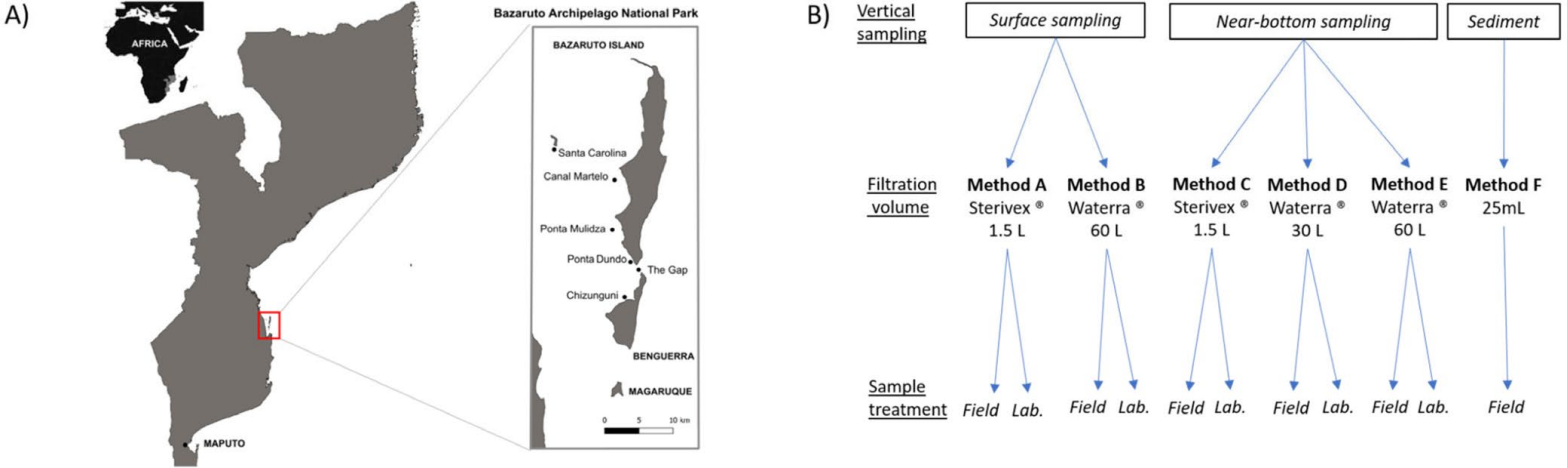
Location of the sampling sites in the Bazaruto Archipelago, Mozambique, and overview of the sampling strategy. (A) Geographic location of the study area and sampling stations within the Bazaruto Archipelago National Park. Black dots indicate sampling sites. (B) Schematic representation of the sampling strategy, illustrating vertical and volume‐based water filtration approaches, followed by DNA extraction treatments performed either in field facilities or in a dedicated environmental DNA laboratory, resulting in a total of 11 distinct sampling–extraction combinations. Lab., Laboratory.

#### 2.1.1 Surface Sampling (Sampling Methods A and B)

Surface sampling was conducted using two different approaches. For the 60 L samples, pre‐pump filtrations were conducted using a diaphragm water pump (Argaly, France) with the cartridge directly submerged from the boat. For the 1.5 L samples, post‐pump filtrations were conducted using a Vampire peristaltic pump (Bürkle, Germany), with an intake hose immersed in a plastic container. This container held a composite sample consisting of 2 L of surface water collected at the beginning and 2 L at the end of each survey track (total volume 4 L), which was thoroughly mixed prior to filtration. This composite sampling design was used to provide a more spatially representative sample of the survey track than a single point collection, thereby improving comparability with the mobile filtration strategies. Two successive filtrations were then performed from the composite sample using separate cartridges, one for each extraction treatment. The plastic container was cleaned with bleach and 70% ethanol, then rinsed with Milli‐Q water at the sampling site prior to each new collection to minimize the risk of cross‐contamination.

#### 2.1.2 Near‐Bottom Sampling (Sampling Methods C–E)

For deeper‐water sampling, two parallel pre‐pump filtrations were conducted using a submersible magnetic drive pump (DroneGo, Mayotte) mounted on a manta tow frame operated by two divers, who ensured that the intake remained approximately 1 m above the seabed while being towed by the boat. Additionally, divers collected 2 L of water at the beginning and 2 L at the end of each survey track in sterile bottles. Once back on the boat, these subsamples were combined and mixed to provide a more spatially representative sample of the survey track, then filtered through successively two filters (2 × 1.5 L), with one filter assigned to each extraction condition (field and laboratory).

#### 2.1.3 Sediment Sampling (Sampling Method F)

At the beginning of each dive, approximately 25 mL of sediment was collected into a graduated 50‐mL sterile tube, with the tube filled to the 25‐mL graduation to standardize the amount of sediment collected across samples.

Samples were preserved in Longmire's buffer (50 mL for Waterra cartridge, 1.5 mL for Sterivex filters, and up to 50 mL for sediment samples). Filtrates were subsequently subjected to vigorous horizontal agitation, performed either manually (sampling methods A, C & F) or using an eDNA Filter Shaker (Waterra, Calgary) (B, D & E), to promote the release of DNA and biological material retained on the filter into the preservation buffer. All samples were stored at approximately 4°C protected from light until DNA extraction. Field extractions were performed on the same day, while the remaining samples were stored at 4°C until shipment for laboratory analysis.

Across the 12 sampling events, the final dataset comprised 107 samples: 20 for strategy A (1.5 L surface; *n* = 13 laboratory extractions/7 field extractions), 21 for B (60 L surface; *n* = 11/10), 17 for C (1.5 L near‐bottom; *n* = 9/8), 19 for D (30 L near‐bottom; *n* = 10/9), 20 for E (60 L near‐bottom; *n* = 10/10), and 10 for F (sediment; field only). Sample sizes differed slightly among strategies because some sampling procedures could not be completed during all sampling events owing to field constraints (Table 1 and more details in Supporting Information).

**TABLE 1:** Overview of sampling events. Depth at each station is indicated in parentheses. Visual observations indicate the timing of dugong sightings relative to sampling. Sampling strategies (A–F) refer to the different water filtration applied during event sampling. A = 1.5 L, surface sampling; B = 60 L, surface sampling; C = 1.5 L, near‐bottom sampling; D = 30 L, near bottom sampling; E = 60 L, near bottom sampling; F = Sediment sampling. Latitude and longitude indicate representative geographic coordinates of each sampling station; sampling was conducted along mobile survey tracks rather than at fixed sampling points.

Sampling event	Date	Start time	Station	Latitude	Longitude	Visual sightings of dugongs	Sampling strategy
1	13/10/2024	10 h 50 min	Ponta Dundo (8 m)	21.79321° S	35.44269° E	At sampling start	B,C,D,E,F
2	13/10/2024	12 h 30 min	Ponta Dundo (8 m)	21.79321° S	35.44269° E	Before sampling (1 h 40 min)	A,B,C,D,E,F
3	13/10/2024	13 h 56 min	Ponta Dundo (8 m)	21.79321° S	35.44269° E	At sampling start	B,C,D,E,F
4	14/10/2024	9 h 50 min	The Gap (5 m)	21.8027° S	35.4664° E	At sampling start	A,B,C,D,E,F
5	14/10/2024	11 h00 min	The Gap (5 m)	21.8027° S	35.4664° E	At sampling start	A,B,C,D,E,F
6	14/10/2024	13 h 30 min	The Gap (5 m)	21.8027° S	35.4664° E	During sampling	A,B,C,D,E,F
7	14/10/2024	15 h 20 min	Ponta_Mulidza (4 m)	21.67278° S	35.41134° E	None	A,B,C,D,E,F
8	18/10/2024	08 h 15 min	Canal Martelo (7 m)	21.65858° S	35.36843° E	During sampling	A,B,C,D,E,F
9	18/10/2024	11 h 15 min	Ponta Dundo (8 m)	21.79321° S	35.44269° E	During sampling	A,B,C,D,E
10	18/10/2024	13 h 00 min	Ponta Dundo (8 m)	21.79321° S	35.44269° E	Before sampling (2 h)	A,B,C,D,E, F
11	14/10/2024	12 h 30 min	Chizunguni (1 m)	21.84541° S	35.43623° E	None	A,B,F
12	17/10/2024	11 h 33 min	Santa Carolina (> 10 m)	21.63241° S	35.33950° E	None	A,B,E

### 2.2 Sample Processing and DNA Extraction

All sampling methods underwent two alternative DNA extraction treatments, carried out either in a dedicated laboratory for low‐concentration and degraded DNA, including environmental DNA samples, or in temporary field facilities located in BANP, except for sediment samples, which were processed exclusively in the field. All DNA extracts were eluted in a volume of 100 μL. In all cases, extraction negative controls were included in each event to monitor potential contamination during the extraction process.

In the field facilities, 5 mL of preservation buffer from each cartridge or sediment sample was divided among four 1.5 mL tubes and centrifuged at 5000 *g* for 20 min. Most of the supernatant was then removed, and the remaining material from the four tubes was re‐pooled for DNA extraction. The field DNA extraction protocol was designed to remain operationally simple under temporary field conditions, and a single versatile extraction procedure using the NucleoSpin eDNA Water kit (Macherey‐Nagel, Düren, Germany), selected for its efficiency and high‐quality DNA yields, was therefore applied across compatible sample types following the manufacturer's recommendations.

In the laboratory, 45 mL Waterra filtrates were centrifuged (15,000 *g* for 15 min) to concentrate organic material (cells, tissues, and free DNA), after which most of the supernatant was removed. DNA from Waterra samples was then extracted using the NucleoSpin eDNA Water kit (Macherey‐Nagel, Düren, Germany), following the manufacturer's recommendations. For Sterivex filters, DNA was extracted using the DNeasy PowerWater Sterivex kit (Qiagen, Hilden, Germany), routinely used in the laboratory for this filter type, following the manufacturer's recommendations.

Overall, “field” and “laboratory” treatments should be understood as two operational processing protocols differing principally in the volume of preservation buffer processed and centrifugation capacity, and, for Sterivex samples, in the extraction kit used.

### 2.3 qPCR and Statistical Analyses

#### 2.3.1 Development of a Specific qPCR Assay

The mitochondrial 16S rRNA gene was selected because of its high copy number and interspecific variability. As a ribosomal gene, it is also more suitable for identifying short specific motifs than more constrained protein‐coding genes. Primer design was based on an alignment of mammalian 16S sequences (see Data S2) using Geneious Prime 2026.0.1 (Kearse et al. 2012). Thermodynamic properties (hairpins, heterodimers, and melting temperature) were assessed using OligoAnalyzer (Integrated DNA Technologies, Coralville, USA). The specificity and efficiency of the qPCR system were assessed using in silico using *ecoPCR* (Ficetola et al. 2010). Primer specificity was further evaluated experimentally by diagnostic PCR, using the Ddug01 primer pair without the TaqMan probe, against DNA from several non‐target vertebrates, including *Chelonia mydas* , *Stenella longirostris* , *Pteropus seychellensis comorensis* , *Bos taurus* , *Mus musculus* and *Homo sapiens* . Analytical performance was further evaluated using serial tenfold dilutions of dugong tissue DNA, with four technical replicates per dilution, to assess amplification linearity, efficiency, and detection performance. The LOD was defined as the lowest DNA concentration reliably detected in all four technical replicates, whereas the LOQ was defined as the lowest concentration remaining sufficiently linear and reproducible for quantitative interpretation (see Data S3).

#### 2.3.2 qPCR Processing

Each sample was amplified in eight technical replicates. The qPCR mix contained 10 μL of Taq360 Gold enzyme mix, 0.16 μL of BSA, 2 μL of each primer (5 μM), 1 μL of TaqMan binding probe (5 μM), and UHQ‐grade water to volume. Potential qPCR inhibition was assessed on a random subset of DNA extracts by comparing amplification profiles of undiluted, 1:2‐diluted, and 1:5‐diluted templates using the Ddug01 assay. Dilution did not improve amplification profiles, which were either similar across dilutions or showed earlier amplification and higher fluorescence plateaus for undiluted extracts, providing no evidence of substantial PCR inhibition. Undiluted DNA was therefore used for all subsequent qPCR analyses, with 2 μL of template DNA added per reaction. Thermal cycling conditions consisted of an initial denaturation at 95°C for 10 min, followed by 60 cycles of 95°C for 30″, 57°C for 30″, and 72°C for 1′, with a final elongation step at 72°C for 7′. A standard curve was generated on each qPCR plate using serial tenfold dilutions (1, 1:10, 1:100, 1:1000) of dugong muscle DNA, run in duplicate. One negative extraction control and one PCR negative control were included in each qPCR run (two replicates each/in duplicate).

### 2.4 Statistical Analysis

For each sample, qPCR detection was expressed as the number of positive qPCR replicates out of eight technical replicates and modeled as a beta‐binomial response (positive vs. negative replicates). In addition, sample‐level detection was summarized as the proportion of samples with at least one positive qPCR replicate for each sampling–extraction strategy, hereafter referred to as the detection rate. Data were analyzed in RStudio (RStudio team 2020) using generalized linear mixed‐effects models with a beta‐binomial distribution (package *glmmTMB*; Brooks et al. 2017). A random intercept for sampling batch was included to account for potential non‐independence among samples collected within the same batch. Fixed effects were sampling volume, vertical position, and sample treatment. The interaction between sampling volume and vertical position was included to account for the dependence between these two sampling factors. Analyses of this interaction were restricted to the fully crossed sampling conditions (1.5 and 60 L; surface and near‐bottom samples; *n* = 78), excluding 30‐L and sediment samples. Significance of fixed effects was assessed using Type II Wald chi‐square tests (package *car*, Fox and Weisberg 2011), and post hoc pairwise comparisons were performed using estimated marginal means (package emmeans; Lenth 2016). Odds ratios (OR) were calculated for pairwise comparisons to quantify the magnitude and direction of differences in qPCR detection between sampling conditions, with OR > 1 indicating higher odds of detection in the first condition relative to the second. Model adequacy was assessed using simulation‐based residual diagnostics (package *DHARMa*; Hartig 2016).

## 3 Results

### 3.1 Development of a Specific qPCR Assay

A primer–probe system named Ddug01 (Dug01F: 5′‐TGC GAG CAG TCC AAA GAA C‐3′; Dug01R: 5′‐GGG ATT AAA TGC TTC TCT TCG‐3′; binding probe: 5′‐CGA GCC CGA TGA TAG CTG GTT G‐3′) was designed to amplify a 115 bp amplicon (Tm = 57°C) located in the 5′ region of the mitochondrial 16S rRNA gene (approx. positions 1–400 of the *Dugong dugon* 16S sequence, GenBank accession PP134992.1). In silico analysis indicated only weak heterodimer formation between the two primers (Δ*G* values ranging from −5.12 to −6.69 kcal/mol), suggesting minimal risk of primer–primer interactions. In silico PCR analysis revealed a potential amplification restricted to *Dugong dugon* and the extinct sirenian *Hydrodamalis gigas* , confirming the assay's high target specificity. Experimental validation using serial tenfold dilutions of dugong tissue DNA showed a consistent amplification response from undiluted DNA through the 10^−4^ dilution (slope = −4.819, *R* ^2^ = 0.984), corresponding to an amplification efficiency of 61.3%. The 10^−4^ dilution, corresponding to 0.944 pg. of DNA per reaction, was consistently detected in all four replicates and was retained as both the limit of detection (LOD) and the limit of quantification (LOQ). Amplification became inconsistent at 10^−5^ (2/4 replicates) and was absent at lower concentrations. A fluorescence threshold of 200 RFU was used to determine Cq values. Detailed calibration results are provided in Data S3.

### 3.2 Field Observation and eDNA Detection

Meteorological conditions were generally homogeneous during the expedition, characterized by clear skies, low precipitation, and calm sea states, which facilitated long‐distance visual observations of dugongs. Except for the Santa Carolina stations, sampling took place during the high‐tide phase of the tidal cycle. Among the 12 survey tracks, dugongs were visually observed at nine locations within 2 h prior to sampling (Table 1 and more details in Data S1). Some sampling events were affected by technical field issues, resulting in the loss or absence of specific sampling strategies. Surface‐water filtration problems during events 1 and 3 prevented collection of the 1.5‐L surface samples (strategy A), while the other water‐sampling strategies were successfully completed. During event 12, diving‐related constraints prevented collection of the 1.5‐ and 30‐L near‐bottom samples (strategies C and D), although the 60‐L near‐bottom sample (strategy E) could be collected. At event 11, the very shallow water depth (< 1 m) precluded meaningful near‐bottom sampling, and only surface‐water and sediment samples (strategies A, B and F) were collected. Finally, technical issues during DNA extraction rendered the sediment samples from events 8–10 unavailable for qPCR analysis. These constraints resulted in unequal sample sizes among sampling strategies (Table 1).

qPCR detection was markedly higher for samples processed using the laboratory protocol than for those processed using the field protocol (Table 2; Figure 2; *χ* ^2^ = 15.06, *p* < 0.001). A significant interaction was detected between filtration volume and vertical sampling position (*χ* ^2^ = 3.87, *p* = 0.049; Table 2). At 1.5 L, near‐bottom sampling significantly increased qPCR detection compared with surface sampling (OR = 5.81, *p* = 0.013; Table 3), corresponding to approximately 5.8‐fold higher odds of a qPCR replicate being positive. In contrast, no difference between vertical positions was detected at 60 L (*p* = 0.810). Conversely, increasing filtration volume from 1.5 to 60 L significantly increased qPCR detection at the surface (OR = 5.49, *p* = 0.012), but not near the bottom (*p* = 0.917; Table 3). Thus, the low detection observed for 1.5‐L samples was primarily associated with surface sampling, whereas at 60 L detection was similar between vertical positions (Figure 2). Model diagnostics supported the adequacy of the beta‐binomial GLMM, with no evidence of significant deviations in residual distribution, dispersion, or zero inflation (See Data S4).

**TABLE 2:** Results of the beta‐binomial generalized linear mixed‐effects model testing the effects of filtration volume, vertical sampling position, sample treatment, and the interaction between filtration volume and vertical position on the probability of a positive qPCR replicate. Sampling batch was included as a random effect. The analysis was restricted to fully crossed sampling conditions (1.5 and 60 L; surface and near‐bottom samples).

Effect	Wald *χ* ^2^	df	Pr(>Chisq)
Sample volume	2.526	1	0.112
Vertical position	2.421	1	0.120
Sample treatment	15.064	1	1.0 × 10^−4^***
Volume × vertical position	3.875	1	0.049*

*Note:* Significance levels: *p < 0.05, ***p < 0.001.

**FIGURE 2:**
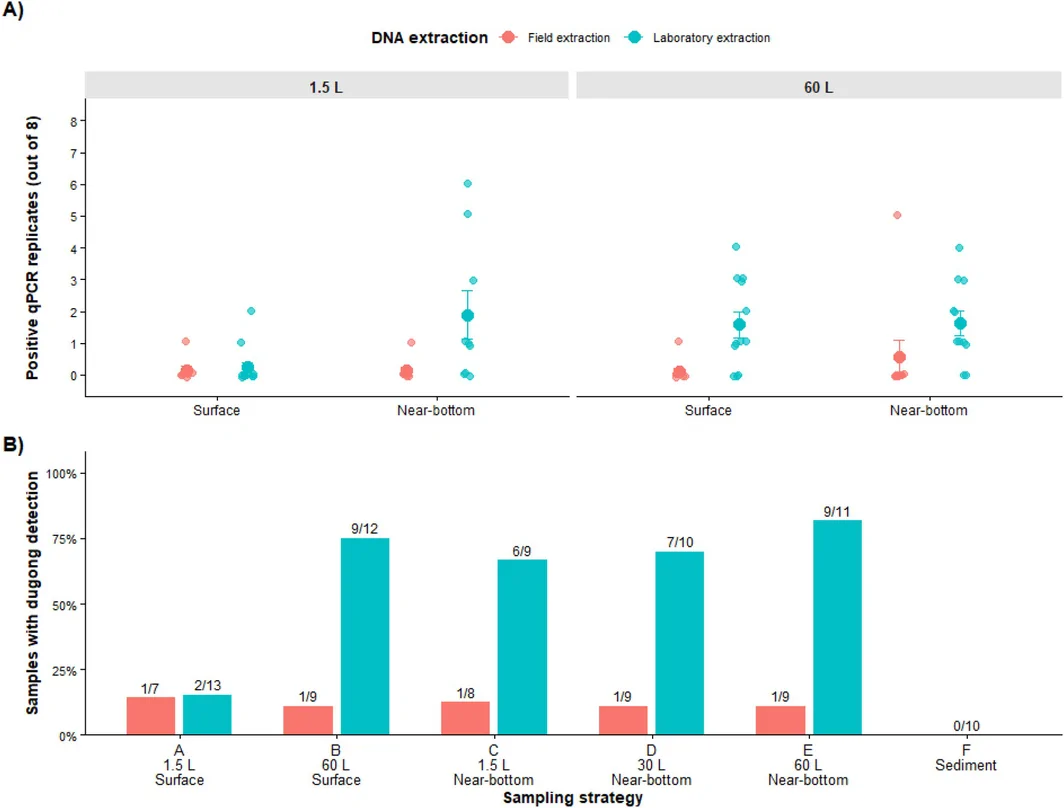
Effects of sampling conditions on dugong eDNA detection. (A) Distribution of the number of positive qPCR replicates per sample (out of eight) for the four sampling conditions included in the beta‐binomial GLMM, according to filtration volume (only for 1.5 or 60 L), vertical sampling position (surface or near‐bottom), and DNA extraction condition (field or laboratory extraction). Individual points represent samples, and larger symbols with error bars indicate mean values and 95% confidence intervals. (B) Detection rate for all sampling strategies, defined as the proportion of samples with at least one positive qPCR replicate. Values above bars indicate the number of detected samples relative to the total number of samples analyzed for each sampling–extraction strategy. Sampling strategies A–F correspond to the combinations of filtration volume and vertical sampling position indicated on the x‐axis. Colors distinguish field and laboratory DNA extraction.

**TABLE 3:** Pairwise comparisons of estimated marginal means from the beta‐binomial GLMM, testing differences in the probability of a positive qPCR replicate between vertical sampling positions within each filtration volume and between filtration volumes within each vertical position. Odds ratios (OR) and *p*‐values are reported.

Pairwise comparison	Odds ratio	*p*
Surface vs near‐bottom at 1.5 L	0.172	0.013*
Surface vs near‐bottom at 60 L	0.896	0.810
1.5 L vs 60 L at surface	0.182	0.0120*
1.5 L vs 60 L near‐bottom	0.949	0.917

*Note:* Significance levels: *p < 0.05.

Overall, dugong DNA was detected in 35% of samples, but sample‐level detection rates were highly heterogeneous among sampling–extraction strategies, following the same general pattern as qPCR detection (Figure 2; see Data S1 for details). At least one positive sample was obtained for each sampling–extraction strategy except sediment sampling. Dugong DNA was detected in samples collected 1 h 40 min and 2 h after the last visual sightings (sampling events 2 and 10, respectively), as well as at sites without direct visual observation (sites 7 and 12). Sampling event 11, during which no dugongs were observed, was the only event with no eDNA detection. The highest sample‐level detection rate was obtained for near‐bottom, large‐volume samples processed in the laboratory (strategy E), with 9 positive samples out of 11.

## 4 Discussion

Results demonstrate that the eDNA‐based approaches successfully detected dugongs in their natural environment. Using the sampling strategy that yielded the highest detection rate (sampling method E combined with laboratory DNA extraction), dugong DNA was detected at all sites where the species had been visually observed up to approximately 2 h prior to sampling. Importantly, positive eDNA detections were also obtained at sites where no dugongs were directly observed.

These observations highlight the complementary temporal and spatial information provided by eDNA and visual surveys, but also the need for caution when interpreting the precise origin of an eDNA signal. First, positive detections may reflect local presence that was not visually detected, since the absence of a sighting cannot be considered evidence of dugong absence, or eDNA persisting locally following an earlier passage. In our study, dugong eDNA remained detectable up to approximately 2 h after the last visual sighting, consistent with killer whale eDNA being detected up to 2 h after animal passage despite substantial displacement of the sampled water mass by tidal currents (Baker et al. 2018). However, the temporal persistence of marine mammal eDNA appears highly context‐dependent: Alter et al. (2022) reported that cetacean eDNA often declined to low levels within 15–30 min following visual sightings, while Robinson et al. (2025) found substantial variation in eDNA detectability through time among cetacean species and individuals. Alternatively, positive detections may originate from eDNA released in adjacent areas and subsequently transported to the sampling location by tidal currents. In this case, eDNA detection would integrate dugong occurrence over a broader spatial footprint than the immediate sampling location, as previously highlighted by the integration of visual surveys, eDNA detection and ocean drift modeling for marine mammals in tropical waters (Nikolic et al. 2025). These mechanisms cannot be distinguished from our data because neither hydrodynamic transport nor the actual presence of undetected dugongs was independently quantified. Positive detections should therefore be interpreted as evidence of recent dugong occurrence within a spatial and temporal window shaped by visual detectability, eDNA persistence and local hydrodynamics, rather than as evidence of the animal being present at the precise location and time of sampling. Conversely, the opposite pattern was observed at a known feeding site, where no dugong eDNA was detected despite the presence of visible feeding trails. As these tracks can persist for a maximum of 20 days in tropical environments (Nakaoka and Aioi 1999; D'Souza et al. 2015), they do not necessarily indicate recent dugong presence. In addition, the strong tidal influence and rapid water renewal at this nearshore site may have accelerated eDNA dilution or removal, potentially contributing to the observed non‐detection (Kelly et al. 2018; Baetscher et al. 2024). This example illustrates that the interpretation of negative eDNA results depends not only on the sensitivity of the sampling and processing workflow, as shown by the differences in detection rates among strategies in our study, but also on the environmental conditions affecting local eDNA availability. Characterizing both components is therefore essential to reduce false negatives and to identify the conditions under which a non‐detection can be more confidently interpreted as evidence of local absence.

The following sections therefore examine how sampling–extraction strategies influence eDNA yield and whether field‐friendly strategies compromise eDNA performance, while interpreting these results in light of the main experimental limitations, particularly confounding among sampling and processing factors and the methodological and logistical constraints inherent to field implementation.

### 4.1 How Do Sampling–Extraction Strategies Influence eDNA Yield?

The recent development of a species‐specific qPCR assay for *D. dugon* further demonstrates the feasibility of targeted eDNA detection for this species (Mousavi‐Derazmahalleh et al. 2026). Importantly, that study identified sampling volume, sampling depth/vertical position, and ecological context as key parameters requiring further optimization. Our field experiment directly addresses two of these methodological dimensions and reveals that filtration volume and vertical sampling position cannot be considered independently. At the low filtration volume (1.5 L), qPCR detection was significantly higher in near‐bottom than in surface samples, whereas this vertical difference was no longer apparent at 60 L. Conversely, the 60‐L strategy showed significantly higher detection than the 1.5‐L strategy at the surface, whereas little difference was observed near the bottom. These results suggest that increasing the sampled water volume can partly compensate for the lower eDNA availability encountered in surface waters, while small‐volume samples remain more sensitive to fine‐scale vertical heterogeneity.

The higher detection observed in near‐bottom samples at low volume is consistent with fine‐scale vertical heterogeneity in dugong eDNA availability within the water column. Such heterogeneity in eDNA concentrations has been widely documented in freshwater systems and can arise from multiple drivers, including patchy spatial distributions of individuals, hydrological dynamics, and water‐column stratification (Jane et al. 2015; Troth et al. 2021; Wood et al. 2021). In coastal marine environments, eDNA dispersal can also be strongly influenced by local hydrodynamic conditions, tidal forcing, temperature, and vertical stratification (Jensen et al. 2022; Baetscher et al. 2024; Nikolic et al. 2025; Silva et al. 2025). For dugongs, one possible explanation is that eDNA release is concentrated near benthic feeding habitats, whereas animals occupy surface waters only briefly for respiration. However, because eDNA concentrations and hydrodynamic conditions were not directly measured, our data cannot distinguish behavioral effects from other processes generating vertical heterogeneity. These findings nevertheless emphasize that sampling depth may strongly influence detection when small water volumes are collected and highlight the value of pilot studies for optimizing species‐specific sampling designs.

Previous eDNA surveys targeting sirenians have generally relied on relatively small water volumes. Hunter et al. (2018) filtered approximately 1 L of water per sample for manatee detection, while the recent dugong‐specific study by Mousavi‐Derazmahalleh et al. (2026) filtered 2 L per replicate. Such small volumes may be particularly sensitive to local vertical and spatial heterogeneity in eDNA availability. In our study, the 60‐L filtration strategy substantially improved detection in surface waters compared with the 1.5‐L strategy, while having little effect near the bottom. However, direct comparisons between the 1.5‐ and 60‐L strategies should be interpreted cautiously, as filtration volume was partly confounded with filter configuration and, in laboratory‐processed samples, extraction kit. Differences in filter properties and DNA extraction procedures may affect DNA capture, recovery, and purity, and differences between these strategies therefore cannot be attributed exclusively to filtration volume. Nevertheless, the smaller pore size was associated with the low‐volume treatment, and the higher detection obtained with the 60‐L strategy despite its larger pore size supports an important contribution of increased sampled volume. Importantly, these methodological differences do not confound the observed vertical pattern, as surface and near‐bottom samples were processed using the same filtration and extraction protocol within each volume treatment. At 60 L, detection probabilities were similar between vertical positions. This suggests that large‐volume filtration can buffer the effect of fine‐scale heterogeneity in eDNA availability. Beyond simply increasing the amount of target DNA captured, mobile large‐volume filtration may integrate eDNA signals over a broader spatial footprint, thereby reducing sensitivity to patchy eDNA distributions. Increased filtration volume may therefore improve detection robustness in heterogeneous marine environments.

No dugong DNA was detected in any of the 10 sediment samples, whereas all water‐based sampling strategies yielded at least some positive detections. This pattern suggests that, under the conditions tested here, sediment sampling may be less effective than water sampling for detecting recent dugong presence. However, sediment samples were processed exclusively in the field, where extraction yielded lower detection performance overall, preventing a direct separation of matrix and extraction effects. Moreover, although the same NucleoSpin eDNA Water kit was successfully used for field extraction of water samples, its efficiency may differ for sediment matrices. The absence of detection could therefore reflect lower eDNA availability in the sampled sediments, matrix‐specific extraction limitations, or a combination of both. Consequently, our results do not demonstrate that sediment is intrinsically unsuitable for dugong eDNA detection, but they provide no evidence of an advantage over water‐based approaches under the operational conditions evaluated here. Sedimentary eDNA may nevertheless integrate biological signals over longer temporal scales than water samples (Sakata et al. 2020; Huston et al. 2023), and may therefore provide information on past rather than recent local presence.

Beyond sampling and processing strategies, detection also depends on molecular assay performance. Ddug01 showed good linearity (*R* ^2^ = 0.984) but relatively low amplification efficiency (61.3%) compared with the recently developed dugong‐specific assay of Mousavi‐Derazmahalleh et al. (2026; 101.8%), potentially reducing sensitivity at low eDNA concentrations. However, this should have limited influence on relative comparisons, as all samples were analyzed under identical conditions. Interestingly, the availability of two dugong‐specific assays with distinct amplification characteristics offers an opportunity for complementary use, potentially reducing primer‐dependent false negatives, as previously shown for intraspecific genetic variants (Corse et al. 2019).

### 4.2 Do Field‐Friendly Strategies Compromise eDNA Performance?

Beyond performance, the 11 sampling–extraction strategies evaluated in this study differed markedly in their field applicability, highlighting trade‐offs between detection sensitivity and operational feasibility. In our study, larger filters provided robust detection while remaining operationally feasible due to the use of positive‐pressure pumps. Using a Waterra cartridge (0.45 μm), filtration was achieved at an average rate of 25 s·L^−1^, allowing 60 L samples to be filtered in less than 25 min, both at the surface and underwater without clogging even at high volumes (up to 100 L in field tests, data not shown), supporting its suitability for high‐volume filtration. These filtration speeds are comparable to those reported by Deeg et al. (2025), who found that optimized capsule‐based systems achieved average filtration rates ranging from 33.1 to 53.9 s·L^−1^, supporting the feasibility of large‐volume filtration when appropriate pump and filter configurations are used. In addition, the pre‐pump filtration configuration limited tubing consumption, a non‐negligible advantage given the high use of single‐use plastics inherent to eDNA sampling. By contrast, peristaltic pump setups placed downstream of the tubing generated substantially more plastic waste, as filters and tubing segments had to be replaced for each sample. Beyond these logistical considerations, field deployment also requires rigorous contamination control throughout sampling and filtration. Although extraction and PCR negative controls showed no amplification, no field or filtration blanks were included in the present study. Consequently, contamination introduced during these upstream steps cannot be formally excluded, and future applications should incorporate such controls.

To combine mobile sampling with filtration at a controlled depth above the seafloor, we adapted a manta tow for near‐bottom eDNA collection along survey tracks, with real‐time depth adjustment facilitated by divers. To our knowledge, eDNA sampling using a towed filtration deployed at a user‐defined depth remains poorly documented, despite the widespread use of towed devices in plankton surveys. This strategy yielded higher eDNA detection than surface sampling at low filtration volume, whereas the difference between vertical positions disappeared at high filtration volume. However, it involved substantially greater logistical constraints, as it required hyperbaric operations. Alternatively, near‐bottom sampling could be achieved using a weighted pumping system (Formel et al. 2021; Hendricks et al. 2023), which would reduce the need for diving activities. However, such an approach would be incompatible with mobile sampling designs conducted along survey tracks, thereby limiting its applicability in dynamic survey protocols. In this context, underwater mobile systems (e.g., Yamahara et al. 2019; Farinha et al. 2022) represent a promising alternative, if compatible with high‐volume filtration.

A strong effect of sample treatment was observed, with field‐extracted samples showing substantially lower qPCR positive rates and detection rates than laboratory‐extracted samples. This effect should be interpreted as a comparison between complete operational processing workflows rather than as an effect of extraction location per se, since the two treatments differed in the volume of preservation buffer processed, centrifugation capacity and, for Sterivex samples, extraction kit. Positive detections indicated that the process did function and that the lower performance reflects a reduction in yield rather than a major technical failure. This reduced efficiency is consistent with the technical constraints encountered during field centrifugation—limited centrifugal force (max 5000 *g*) and the inability to process large volumes of preservation buffer—which likely lowered the amount of recoverable DNA. Such conditions can pellet intact cells but are less effective for recovering free or weakly adsorbed DNA, which typically requires higher centrifugal forces or precipitation steps involving reagents such as ethanol, CTAB–chloroform, or phenol–chloroform (e.g., Pembrey et al. 1999). These reagents, however, are impractical to use on site due to their toxicity, volatility, and transport restrictions. Therefore, mitigating constraints associated with limited centrifugal force and field‐incompatible DNA precipitation remains a key challenge for reliable eDNA extraction in remote, logistically constrained environments.

## 5 Conclusion

Overall, our results demonstrate the feasibility of eDNA‐based dugong detection and show that sampling volume and vertical position jointly influence detection performance. At low filtration volume, near‐bottom sampling substantially improved detection, whereas increasing filtration volume improved surface detection and largely removed differences between vertical sampling positions. For large‐scale dugong monitoring, our results therefore support mobile surface sampling combined with high‐volume filtration as a practical approach, providing a favorable compromise between detection performance and operational scalability. In particular, surface sampling does not require divers or the deployment of additional submerged equipment, substantially reducing the logistical requirements associated with large‐scale surveys. In addition, the pre‐pump configuration used for high‐volume filtration limited tubing consumption compared with the post‐pump configuration used for low‐volume filtration, representing an operational advantage in terms of single‐use consumables. Such mobile sampling could be targeted toward areas of suspected dugong use, particularly seagrass habitats, thereby increasing the spatial coverage of suitable habitats. When high‐volume filtration is not feasible, targeting near‐bottom waters may improve detection performance, particularly for low‐volume sampling, although this approach involves greater logistical constraints. Given the limited number of sampling events and positive detections in this proof‐of‐concept study, extending the sampling effort across broader spatial, temporal, and hydrodynamic conditions, particularly across tidal cycles, will now be important to determine whether survey timing can improve detection probability, better characterize the spatio‐temporal window represented by dugong eDNA, and assess the general applicability of the proposed sampling framework. Ultimately, such an approach could facilitate the integration of eDNA into monitoring programs for small and difficult‐to‐observe dugong populations, particularly across extensive tropical coastal habitats where conventional surveys remain challenging.

## Author Contributions

**Lea Bernagou:** conceptualization (equal), funding acquisition (equal), investigation (equal), project administration (lead), writing – original draft (equal). **Auriane Serval:** conceptualization (equal), investigation (equal), project administration (equal), writing – review and editing (equal). **François Beudard:** conceptualization (equal), funding acquisition (equal), investigation (equal), project administration (supporting). **Jerome Mathey:** investigation (equal), methodology (equal), resources (equal), writing – review and editing (equal). **Yann Mercky:** investigation (equal), methodology (equal), writing – original draft (equal). **Charles Le Bozec:** investigation (equal), methodology (equal), writing – original draft (equal). **Evan Trotzuk:** investigation (equal), project administration (supporting), writing – review and editing (equal). **Keila Paunde:** investigation (equal), writing – review and editing (equal). **Marielle Garcia:** data curation (equal), methodology (equal), visualization (equal), writing – original draft (equal). **Eva Bellemain:** data curation (equal), methodology (equal), resources (equal), validation (equal), writing – original draft (equal). **Emmanuel Corse:** conceptualization (equal), formal analysis (equal), investigation (equal), methodology (equal), writing – original draft (lead), writing – review and editing (lead).

## Funding

This work was supported by European Regional Development Fund (ERDF), INTERREG Indian Ocean Programme (Phase V); Interreg PO Mozambique Channel (Phase VI).

## Conflicts of Interest

The authors declare no conflicts of interest.

## Supporting information


**Data S1:** Sampling metadata and eDNA detection results. Mean Cq values correspond to the average quantification cycle values calculated from technical replicates that showed successful amplification during qPCR.

**Data S2:** Alignment of vertebrate 16S gene sequences from GenBank used for the design of dugong‐specific qPCR primers. FASTA file containing an alignment of 16S rRNA gene sequences from multiple vertebrate taxa, including the primer–probe system developed in this study for *Dugong dugon* . Sequence headers include species names and corresponding GenBank accession numbers. The alignment was used to identify conserved and variable regions to guide the design of the dugong‐specific qPCR assay.

**Data S3:** Analytical validation and performance of the Ddug01 qPCR assay. Results of serial dilution experiments used to assess amplification linearity, qPCR efficiency, detection performance at low DNA concentrations, and analytical limits (LOD and LOQ). The file includes the standard curve, replicate‐level detection patterns, detailed amplification results, and a summary of analytical performance parameters.

**Data S4:** Statistical model validation and model selection. Diagnostic assessment of the final beta‐binomial GLMM, including simulation‐based residual diagnostics, tests for dispersion, zero inflation and outliers, and comparison with alternative model structures incorporating additional two‐way and three‐way interactions.

## Data Availability

All data underlying the main results are provided in Supporting File II accompanying the manuscript. Primer and probe sequences for the species‐specific qPCR assay are reported in the main text.
