# Supplementary material for: Optimizing Marine eDNA Protocols for Rare Megafauna: A Case Study on Dugongs in the Western Indian Ocean
Source: Ecol Evol. 2026 Oct 16;16(10):e74500. doi: 10.1002/ece3.74500 (PMC13675653; doi:10.1002/ece3.74500)
Supplement: Supplementary file 1 [file ECE3-16-e74500-s002.docx]

**Supplementary Information III**

**Analytical validation and performance of the Ddug01 qPCR assay**

Additional validation experiments were performed to further characterize the analytical performance of the Ddug01 qPCR assay. Serial tenfold dilutions of dugong DNA were used to assess amplification linearity, qPCR efficiency, and detection performance at low DNA concentrations, and to determine the limit of detection (LOD) and limit of quantification (LOQ). The standard curve and the proportion of positive qPCR replicates across the dilution series are presented in Figure 1. Detailed amplification results for each dilution, including the number of positive replicates and Cq values, are provided in Table 1, while the main analytical performance parameters of the assay are summarized in Table 2.

**Figure 1. Analytical validation of the Ddug01 qPCR assay.** *(A) Standard curve obtained from serial tenfold dilutions of dugong DNA. The regression was fitted over the dilution range from undiluted DNA (1) to 10⁻⁴, with individual qPCR replicates shown as points. The resulting regression had a slope of −4.819, an R² of 0.984, and an amplification efficiency of 61.3%. (B) Proportion of positive qPCR replicates across the complete dilution series (1 to 10⁻⁹). Labels indicate the number of positive replicates relative to the four replicates tested at each dilution. All four replicates were positive down to 10⁻⁴, two of four were positive at 10⁻⁵, and no amplification was detected from 10⁻⁶ to 10⁻⁹. The dashed line indicates the LOD/LOQ at 10⁻⁴, corresponding to 0.944 pg of DNA per reaction.*

| **DNA dilution** | **Replicates tested** | **Positive replicates** | **Detection rate** | **Mean Cq ± SD** |
| --- | --- | --- | --- | --- |
| 1 | 4 | 4 | 100% | 17.17 ± 0.09 |
| 10⁻¹ | 4 | 4 | 100% | 21.04 ± 0.02 |
| 10⁻² | 4 | 4 | 100% | 25.21 ± 0.07 |
| 10⁻³ | 4 | 4 | 100% | 29.89 ± 0.20 |
| 10⁻⁴ | 4 | 4 | 100% | 36.83 ± 0.55 |
| 10⁻⁵ | 4 | 2 | 50% | 46.77 ± 11.08 |
| 10⁻⁶ | 4 | 0 | 0% | – |
| 10⁻⁷ | 4 | 0 | 0% | – |
| 10⁻⁸ | 4 | 0 | 0% | – |
| 10⁻⁹ | 4 | 0 | 0% | – |

**Table 1. Amplification performance of the Ddug01 qPCR assay across the serial dilution series**. *Four technical replicates were tested at each DNA dilution. Detection rate corresponds to the proportion of replicates showing positive amplification. Mean Cq and standard deviation were calculated from positive replicates only.*

| **Parameter** | **Value** |
| --- | --- |
| Dilution range used for standard curve | 1–10⁻4 |
| Slope | −4.819 |
| R² | 0.984 |
| qPCR efficiency | 61.3% |
| LOD | 10⁻⁴ (0.944 pg/reaction) |
| LOQ | 10⁻⁴(0.944 pg/reaction) |
| Replicates at LOD/LOQ | 4/4 positive |
| Detection at 10⁻⁵ | 2/4 positive |
| Detection at ≤10⁻⁶ | 0/4 positive |

**Table 2. Summary of the analytical performance of the Ddug01 qPCR assay***. Regression parameters and amplification efficiency were calculated over the dilution range from undiluted DNA to 10⁻⁴. The LOD and LOQ were both retained at 10⁻⁴, corresponding to 0.944 pg of DNA per reaction. This dilution was the lowest concentration reliably detected in all four technical replicates (LOD) and the lowest concentration remaining sufficiently linear and reproducible for quantitative interpretation (LOQ).*
