# Supplementary material for: Optimizing Marine eDNA Protocols for Rare Megafauna: A Case Study on Dugongs in the Western Indian Ocean
Source: Ecol Evol. 2026 Oct 16;16(10):e74500. doi: 10.1002/ece3.74500 (PMC13675653; doi:10.1002/ece3.74500)
Supplement: Supplementary file 2 [file ECE3-16-e74500-s004.docx]

**Supplementary Information IV**

**Statistical model validation and model selection**

To assess the robustness of the statistical analysis of qPCR detection, the adequacy and structure of the final beta-binomial GLMM were evaluated. Model fit was assessed using simulation-based residual diagnostics, presented graphically in Figure 1 and summarized in Table 1. Alternative model structures incorporating additional interactions among sampling variables were then compared to support selection of the final model (Table 2).

**Figure 1 Diagnostic plots of the final beta-binomial generalized linear mixed-effects model.** *Simulation-based residual diagnostics were performed using the DHARMa package. (A) Quantile–quantile plot of simulated residuals showing no significant deviation from the expected distribution (Kolmogorov–Smirnov test, P = 0.775), no evidence of over- or underdispersion (P = 0.776), and no significant outliers (P = 1.000). (B) DHARMa residuals plotted against model predictions, showing no significant systematic deviation of residuals across fitted values. Overall, the diagnostics indicated an adequate fit of the beta-binomial GLMM used to analyze qPCR detection.*

| **Diagnostic** | **Statistic / result** | **P** |
| --- | --- | --- |
| **Residual uniformity (KS test)** | D = 0.073 | 0.775 |
| **Dispersion** | 1.054 | 0.776 |
| **Zero inflation** | Obs./sim. ratio = 0.981 | 0.860 |
| **Outliers** | No deviation detected | 1.000 |

**Tableau 1 Model diagnostics for the final beta-binomial generalized linear mixed-effects model.** *Simulation-based residual diagnostics were performed using the DHARMa package. Tests assessed residual uniformity, dispersion, zero inflation, and the presence of outliers. No significant deviation was detected for any diagnostic, supporting the adequacy of the final model.*

| **Model** | **Additionnal interactions** | **AIC** | **LRT P** |
| --- | --- | --- | --- |
| **Final model** | Volume × Vertical position | 176.35 | _ |
| **Two-way interaction model** | + Volume × Treatment + Vertical position × Treatment | 179.48 | 0.650 |
| **Full model** | + Volume × Vertical position × Treatment | 180.23 | 0.262 |

**Tableau 2 Comparison of beta-binomial generalized linear mixed-effects models used to evaluate interaction terms.** *The final model included the interaction between filtration volume and vertical sampling position, with sample treatment as an additive fixed effect and sampling batch as a random effect. More complex models additionally included interactions between sample treatment and the sampling variables. Model fit was compared using Akaike’s Information Criterion (AIC) and likelihood-ratio tests (LRT). Lower AIC values indicate greater support for the model while accounting for model complexity. ¹ LRT comparison with the model containing all two-way interactions (Volume × Vertical position, Volume × Sample treatment, and Vertical position × Sample treatment).*
